# WIPI-1 Positive Autophagosome-Like Vesicles Entrap Pathogenic *Staphylococcus aureus* for Lysosomal Degradation

**DOI:** 10.1155/2012/179207

**Published:** 2012-07-09

**Authors:** Mario Mauthe, Wenqi Yu, Oleg Krut, Martin Krönke, Friedrich Götz, Horst Robenek, Tassula Proikas-Cezanne

**Affiliations:** ^1^Autophagy Laboratory, Interfaculty Institute for Cell Biology, Eberhard Karls University Tübingen, Auf der Morgenstelle 15, 72076 Tübingen, Germany; ^2^Microbial Genetics, Interfaculty Institute for Microbiology and Infectious Medicine, University of Tübingen, 72076 Tübingen, Germany; ^3^Institute for Medical Microbiology, Immunology and Hygiene, University of Cologne, 50935 Cologne, Germany; ^4^Leibniz Institute for Arteriosclerosis Research, University of Münster, 48149 Münster, Germany

## Abstract

Invading pathogens provoke the autophagic machinery and, in a process termed xenophagy, the host cell survives because autophagy is employed as a safeguard for pathogens that escaped phagosomes. However, some pathogens can manipulate the autophagic pathway and replicate within the niche of generated autophagosome-like vesicles. By automated fluorescence-based high content analyses, we demonstrate that *Staphylococcus aureus* strains (USA300, HG001, SA113) stimulate autophagy and become entrapped in intracellular PtdIns(3)P-enriched vesicles that are decorated with human WIPI-1, an essential PtdIns(3)P effector of canonical autophagy and membrane protein of both phagophores and autophagosomes. Further, *agr*-positive *S. aureus* (USA300, HG001) strains were more efficiently entrapped in WIPI-1 positive autophagosome-like vesicles when compared to *agr*-negative cells (SA113). By confocal and electron microscopy we provide evidence that single- and multiple-Staphylococci entrapped undergo cell division. Moreover, the number of WIPI-1 positive autophagosome-like vesicles entrapping Staphylococci significantly increased upon (i) lysosomal inhibition by bafilomycin A_1_ and (ii) blocking PIKfyve-mediated PtdIns(3,5)P_2_ generation by YM201636. In summary, our results provide evidence that the PtdIns(3)P effector function of WIPI-1 is utilized during xenophagy of *Staphylococcus aureus*. We suggest that invading *S. aureus* cells become entrapped in autophagosome-like WIPI-1 positive vesicles targeted for lysosomal degradation in nonprofessional host cells.

## 1. Introduction

Macroautophagy (hereafter autophagy) is a cytoprotective cellular degradation mechanism for long-lived proteins and organelles [1]. Autophagy is specific to eukaryotic cells and important for cellular survival by enabling a constitutive clearance and recycling of cytoplasmic material (basal autophagy). Crucial to the process of autophagy is the fact, that cytoplasmic material is stochastically degraded. Portions of the cytoplasm become randomly sequestered in unique, double-membrane vesicles, autophagosomes. Autophagosomes are generated by elongation and closure of a membrane precursor, the phagophore. Subsequently, autophagosomes fuse with lysosomes to acquire acidic hydrolases for cargo degradation [2]. This stochastic constitutive form of autophagy provides constant clearance of the cytoplasm. Upon stress, such as starvation, the autophagic activity is induced above basal level to compensate nutrient shortage by providing monomeric constituents, such as amino acids, and energy. Conversely, under nutrient-rich conditions autophagy is suppressed by the mTORC1 signaling circuit [3]. Importantly, autophagy is also activated in a specific manner and targets damaged organelles, protein aggregates, or pathogens for degradation [4]. Both, stochastic and specific autophagy are crucial to secure cellular homeostasis [5].

Prerequisite for the formation of autophagosomes is the generation of an essential phospholipid, phosphatidylinositol 3-phosphate (PtdIns(3)P), a result of the activity of the phosphatidylinositol 3-kinase class III (PtdIns3KC3) in complex with Beclin 1, p150, and Atg14L [6, 7]. The PtdIns(3)P signal is decoded through PtdIns(3)P-binding effectors specific to autophagy, such as the human WIPI proteins [8]. WIPI-1 (Atg18 in yeast) specifically binds PtdIns(3)P at the phagophore and fosters the recruitment of two ubiquitin-like conjugation systems, Atg12 and LC3, involved in phagophore elongation and closure [9]. Subsequently, WIPI-1 becomes a membrane protein of autophagosomes where it localizes at both the inner and outer membrane [10, 11]. Hence the specific localization of WIPI-1 at the phagophore and at autophagosomes upon the initiation of autophagy can monitor the process of canonical autophagy, as it is dependent on the PtdIns(3)P signal [11].

The process of autophagy is closely connected with a variety of diseases such as tumor development, neurodegeneration, and with cellular responses to pathogens, including viral infection and bacterial cell invasion [5, 12]. *Staphylococcus aureus*, a major pathogen for nosocomial infectious diseases was initially characterized as an extracellular pathogen, but was later found to also target nonprofessional host cells like keratinocytes, fibroblasts, endothelial cells, and epithelial cells where invading *S. aureus* liberates from the endosomal compartment [13]. In HeLa cells, *S. aureus* was found to become sequestered and to replicate in autophagosome-like vesicles as a result of autophagosome/lysosome fusion block, which ultimately leads to cell death [14].

Here, we visualized the invasion of mCherry-expressing *S. aureus* strains USA300, HG001, SA113 in human U2OS tumor cells that stably express GFP-WIPI-1 for automated fluorescence-based high content analyses, a procedure that monitors the autophagic process and that we have established earlier [15]. We provide evidence that *S. aureus* stimulates canonical autophagy in nonprofessional host cells and becomes entrapped in noncanonical WIPI-1 positive autophagosome-like vesicles. Time course experiments showed that the number of tumor cells that contain such WIPI-1 positive autophagosome-like vesicles with entrapped *S. aureus* cells increased over time (30 min–2 h). After an infection period of 2 h, 40–50% of the cells harbored WIPI-1 positive autophagosome-like vesicles sequestering *agr*-positive *S. aureus* (USA300, HG001), and 20% of the tumor cells contained entrapped *agr*-negative *S. aureus* (SA113). Importantly, we demonstrate that the number of WIPI-1 positive autophagosome-like vesicles harboring *S. aureus* significantly increased upon lysosomal inhibition, strongly arguing for the degradation of *S. aureus* through xenophagy. In addition, by employing GFP-FYVE and a selective PIKfyve inhibitor (YM201636) we further demonstrate the requirement of PtdIns(3)P-enriched membranes during the process of entrapping invading *S. aureus*.

## 2. Material and Methods

### 2.1. Eukaryotic Cell Culture

The human osteosarcoma cell line U2OS (ATCC) was cultured in DMEM (Invitrogen) supplemented with 10% FCS (PAA), 100 U/mL penicillin/100 *μ*g/mL streptomycin (Invitrogen), 5 *μ*g/mL plasmocin (Invivogen) at 37°C, 5% CO_2_. Monoclonal human U2OS cell clones stably expressing either GFP-WIPI-1 [15, 20] or GFP-2xFYVE [9] were cultured in DMEM (Invitrogen) supplemented with 10% FCS (PAA), 100 U/mL penicillin/100 *μ*g/mL streptomycin (Invitrogen), 5 *μ*g/mL plasmocin (Invivogen), 0.6 mg/mL G418 (Invitrogen) at 37°C, 5% CO_2_. The following media were used for treatments: DMEM/FCS (DMEM supplemented with 10% FCS), DMEM (DMEM without FCS), and EBSS (Sigma-Aldrich).

### 2.2. Bacterial Strains

*S. aureus* USA300, HG001, SA113, or *S. carnosus* TM300 [21] (see Table 1) were electroporated with the pCtuf-*ppmch* plasmid. The pCtuf-*ppmch* plasmid encoded mCherry fused with the propeptide of lipase for fluorescence enhancement, and *ppmch* expression was controlled by the native constitutive EF-Tu promotor. Electroporated bacterial strains were grown in basic medium (1% peptone, 0.5% yeast extract, 0.5% NaCl, 0.1% glucose, 0.1% K_2_HPO_4_) at 37°C to an OD_600_ of 0.8 and harvested by centrifugation.

### 2.3. Bacterial Infection of Eukaryotic Host Cells

GFP-WIPI-1 expressing U2OS cells were seeded in 96-well plates (Brand) in DMEM/10% FCS 20 hours before bacterial infection. *S. aureus* (USA300, HG001, SA113) or *S. carnosus* carrying the pCtuf-*ppmch* plasmid, were diluted in DMEM, DMEM/10% FCS or EBSS (Sigma-Aldrich) to an m.o.i of 100, added to the GFP-WIPI-1 U2OS cells, and incubated for 0.5, 1, or 2 hours at 37°C, 5% CO_2_. Alternatively, *S. aureus* USA300 cells were diluted (m.o.i of 100) in DMEM/FCS supplemented with either bafilomycin A_1_ (200 nM, Sigma-Aldrich) or YM201636 (800 nM, Cayman Chemicals) or with both and used to infect GFP-WIPI-1 expressing U2OS cells for 2 hours at 37°C, 5% CO_2_. Alternatively, GFP-2xFYVE expressing U2OS cells [9] were infected with *S. aureus* USA300 (in DMEM/FCS) for 2 hours at 37°C, 5% CO_2_.

### 2.4. Autophagy Assay

GFP-WIPI-1 expressing U2OS cells, seeded in 96-well plates, were treated with nutrient-rich culture medium (DMEM/10% FCS), culture medium lacking serum (DMEM), or medium lacking serum and amino acids (EBSS) for 0.5, 1, or 2 hours. After fixation with 3.7% paraformaldehyde for 30 minutes, autophagy was accessed by WIPI-1 puncta formation analysis [11, 22] (see below).

### 2.5. Confocal Laser Scanning Microscopy

Confocal microscopy was conducted as previously described [8]. Images were acquired using an LSM510 microscope (Zeiss) and a 63 × 1.4 DIC Plan-Apochromat oil-immersion objective. For each image, 8–10 optical sections (0.5 *μ*m) were acquired. Both, single optical sections as well as projections from 8–10 optical sections are presented.

### 2.6. Automated Fluorescence Image Acquisition and Analysis

Stable GFP-WIPI-1 U2OS cells were automatically imaged and analysed using the* In Cell Analyzer 1000* (GE Healthcare) as described earlier [9, 15]. Cells exposed to bacteria (see above) were stained with DAPI (5 *μ*g/mL; Applichem). Fluorescence images were automatically acquired with a Nikon 40x Plan Fluor objective and the excitation/emission filter D360_40X/HQ460_40M (DAPI), HQ535_50X/HQ620_60 M (mCherry), and S475_20X/HQ535_50M (GFP). GFP-WIPI-1 puncta were automatically analysed as previously described [15] and the number of GFP-WIPI-1 puncta-positive cells as well as the number of GFP-WIPI-1 puncta per cell was determined. Red fluorescent bacteria were automatically analysed by using the *dual area object analysis*. The algorithms *inclusion* and *multiscale top hat* were applied and the total area of bacterial fluorescence within the cell was determined. To determine the number of cells containing GFP-WIPI-1 positive autophagosome-like vesicles sequestering bacteria, automatically acquired fused images (DAPI, GFP, mCherry) of 100 individual cells for each treatment were analyzed.

### 2.7. Electron Microscopy

Stable GFP-WIPI-1 U2OS cells were infected with *S. aureus* USA300 (m.o.i of 100) in DMEM/FCS and fixed in 2% glutaraldehyde and 0.5% osmium tetroxide in 0.1 M PBS, dehydrated with ethanol, and embedded in Epon using standard procedures as previously described [23]. Thin sections were cut using an ultramicrotome and contrasted with uranyl acetate and lead citrate. Thin sections were examined in an EM410 electron microscope (Philips) and documented digitally (DITABIS).

### 2.8. Statistical Analysis

Statistical significance was evaluated using two-tailed heteroscedastic *t*-testing and *P* values were calculated.

## 3. Results

### 3.1. Visualizing Basal and Induced Autophagy by Automated GFP-WIPI-1 Image Acquisition and Analysis

**Figure 1:**
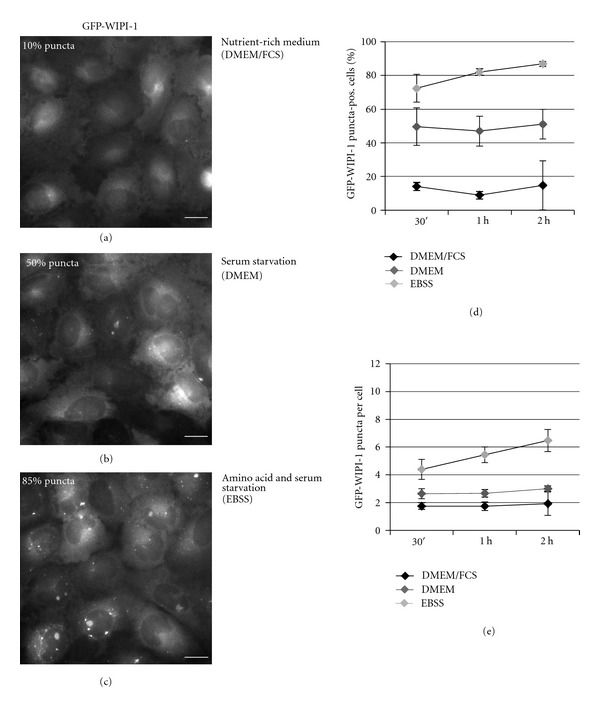
GFP-WIPI-1 puncta formation upon serum and amino acid starvation. GFP-WIPI-1 U2OS cells were treated with nutrient-rich culture medium (DMEM/FCS), serum-free culture medium (DMEM), or with medium lacking both serum and amino acids (EBSS) for 0.5, 1, and 2 h. Fluorescence images were automatically acquired and 2 h treatment images are shown ((a)–(c)). The number of GFP-WIPI-1 puncta-positive cells (d), and of GFP-WIPI-1 puncta per cell (e) was automatically determined. Each measure point represents mean value from up to 3000 individually analyzed cells per treatment condition ± SD (*n* = 2, each in triplicates). Scale bars: 20 *μ*m.

The WIPI-1 puncta-formation assay allows the assessment of the evolutionarily conserved, PtdIns(3)P-dependent initiation of autophagy on the basis of fluorescence microscopy, previously employed by using confocal microscopy or automated image acquisition and analysis [11, 15]. Thereby, endogenous WIPI-1 can be visualized by indirect immunofluorescence or alternatively by introducing GFP-WIPI-1 as conducted in the present study. Fluorescent WIPI-1 puncta reflect the accumulation of WIPI-1 at membranes via its specific binding to PtdIns(3)P was found to represent phagophores and autophagosomes [10, 11]. In addition, WIPI-1 binds to PtdIns(3)P at the endoplasmic reticulum and at the plasma membrane upon the induction of autophagy, indicative for membrane origins where phagophore/autophagosome formation is initiated by unknown mechanisms [10]. Here, we employed automated GFP-WIPI-1 image acquisition and analysis as follows. Human U2OS cells that stably express GFP-WIPI-1 were seeded in 96-well plates and basal autophagy, and starvation-induced autophagy was monitored in up to 3000 individual cells per treatment over time (Figure 1). After an incubation period of 0.5, 1, or 2 h with nutrient-rich culture medium (DMEM/FCS), basal autophagic activity was found in approximately 10% of the cells (Figures 1(a) and 1(d)). Serum starvation (DMEM) elevated the number of GFP-WIPI-1 puncta-positive cells to approximately 50% (Figures 1(b) and 1(d)), and both serum and amino acid starvation (EBSS) further elevated this number to approximately 85% (Figures 1(c) and 1(d)). In addition, we demonstrate that with regard to nutrient-rich medium (DMEM/FCS), the number of GFP-WIPI-1 puncta per cell also increased upon serum (DMEM) or upon both serum and amino acid starvation (EBSS) (Figure 1(e)). These culture media (DMEM/FCS, DMEM, EBSS) were used in the following experiments to infect GFP-WIPI-1 expressing U2OS cells with mCherry-expressing Staphylococci.

### 3.2. Formation of GFP-WIPI-1 Positive Autophagosome-Like Vesicles upon *Staphylococcus aureus* Infection

**Figure 2:**
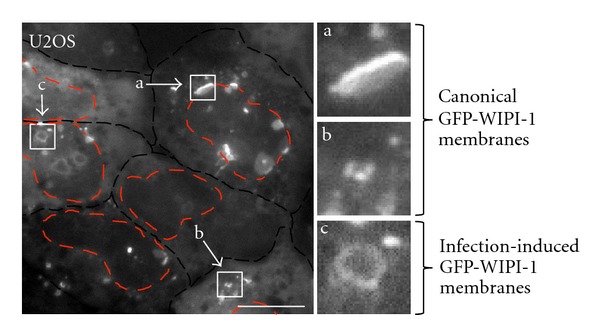
GFP-WIPI-1 images upon infection of U2OS cells with *S. aureus *HG001. GFP-WIPI-1 U2OS cells were infected with *S. aureus *HG001 in DMEM/FCS for 2 h and images were automatically acquired. GFP-WIPI-1 fluorescence of the cells (indicated with the black-dashed line) is shown, and cell nuclei are indicated (red-dashed line) according to DAPI staining (not shown). Highlighted are the different GFP-WIPI-1 structures observed: large perinuclear GFP-WIPI-1 positive membranes (a) and cytoplasmic GFP-WIPI-1 puncta (b), reflecting canonical autophagosomal membranes. In addition, GFP-WIPI-1 positive autophagosomal-like vesicles appeared specifically upon infection (c). Scale bars: 20 *μ*m. Supplementary information is provided (Supplementary Figure  1).

Upon infection of GFP-WIPI-1 U2OS cells with pathogenic Staphylococci, here *S. aureus* HG001, in nutrient-rich medium (DMEM/FCS), we identified canonical, autophagosomal GFP-WIPI-1 membranes (Figures 2(a) and 2(b)), and new GFP-WIPI-1 autophagosome-like vesicles that were larger in diameter with decreased fluorescence intensity (Figure 2(c)) when compared to the canonical GFP-WIPI-1 puncta. GFP-WIPI-1 autophagosome-like vesicles (Figure 2(c)) were rarely observed when starvation media (DMEM, EBSS) were used during the infection with *S. aureus* HG001 (Supplementary Figure  1 available online at doi:10.1155/2012/179207).

**Figure 3:**
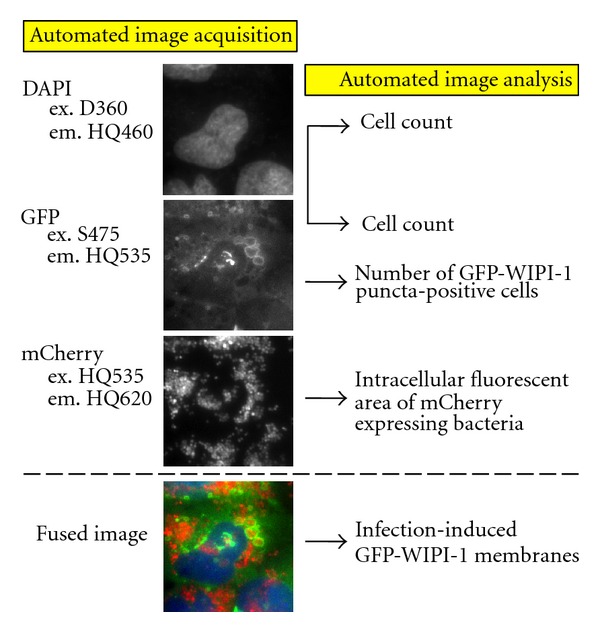
Automated image acquisition and analysis of stably expressing GFP-WIPI-1 U2OS cells with mCherry-expressing Staphylococci. Fluorescence images (middle panel) were automatically acquired using different emission/excitation filters for DAPI, GFP, and mCherry (left panel). DAPI and GFP images were used to automatically detect individual cells, and GFP images were used for detecting and analyzing GFP-WIPI-1 puncta formation (indicated in the right panel). Additionally, for each individual cell the bacterial area was determined (indicated in the right panel) and a fused image was further used to determine the number of cells harboring WIPI-1 positive autophagosome-like vesicles entrapping Staphylococci.

To monitor and quantify this particular GFP-WIPI-1 response upon mCherry-expressing Staphylococci infection in an automated fashion (Figure 3), cells were stained with DAPI and by using three different excitation/emission filters, DAPI, GFP, and mCherry fluorescence images were acquired (Figure 3). Up to 2723 individual cells per treatment were automatically recognized by both DAPI and the overall cellular GFP fluorescence. GFP images were used to automatically detect and determine the number of cells harboring GFP-WIPI-1 puncta by applying a decision tree as previously described [15]. Additionally, mCherry fluorescence was used to automatically determine the fluorescence area, reflecting the load of intracellular Staphylococci. For the quantification of cells harboring GFP-WIPI-1 positive autophagosome-like vesicles entrapping Staphylococci, fused images (DAPI, GFP, mCherry) of 100 individual cells were used (Figure 3).

### 3.3. Pathogenic *Staphylococcus aureus* USA300, HG001, and SA113 Stimulated Canonical Autophagosome Formation and Became Entrapped in GFP-WIPI-1 Positive Autophagosome-Like Vesicles

**Figure 4:**
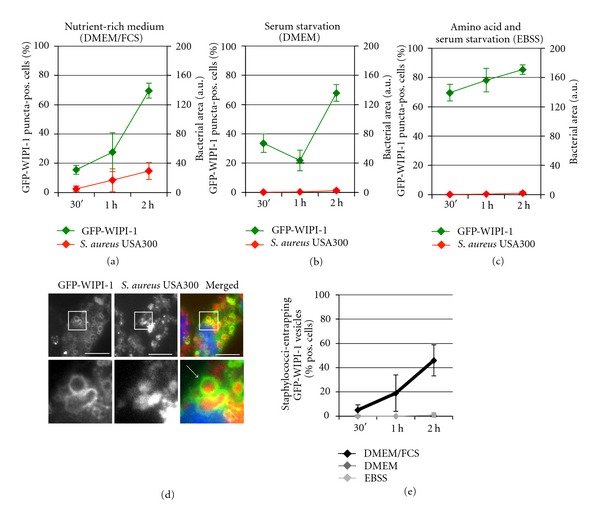
Pathogenic *S. aureus* USA300 induces GFP-WIPI-1 puncta formation and becomes entrapped in GFP-WIPI-1 positive autophagosome-like vesicles. GFP-WIPI-1 U2OS cells were infected with mCherry-expressing *S. aureus* USA300 for 0.5, 1, and 2 h in DMEM/FCS, DMEM, or EBSS. Automated image acquisition and analysis were conducted as described in Figure 3. The quantification of up to 2000 individual cells is presented for GFP-WIPI-1 (in green) and *S. aureus* USA300 (in red) using either DMEM/FCS (a), DMEM (b), or EBSS (c) for infection ± SD (*n* = 2, each in duplicates). Representative images (2 h infection in DMEM/FCS) are shown (d). Scale bars: 20 *μ*m. From 100 infected cells for each of the treatment condition, the number of cells displaying GFP-WIPI-1 positive autophagosomal-like vesicles entrapping *S. aureus* USA300 was determined (e) ± SD (*n* = 2, each in duplicates).

**Figure 5:**
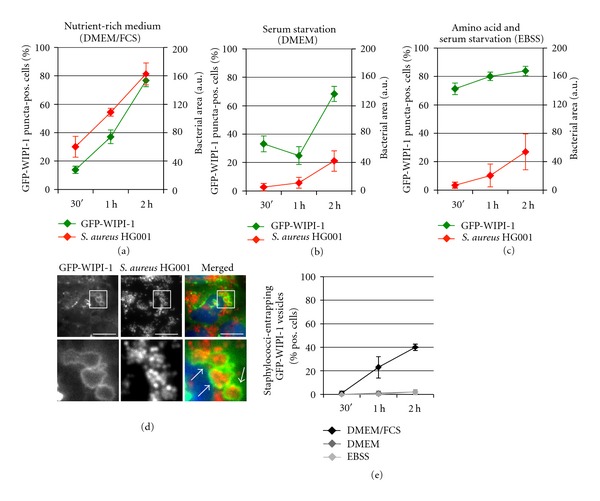
Pathogenic *S. aureus* HG001 induces GFP-WIPI-1 puncta formation and becomes entrapped in GFP-WIPI-1 positive autophagosome-like vesicles. According to Figure 4, GFP-WIPI-1 U2OS cells were infected with mCherry-expressing *S. aureus* HG001 in DMEM/FCS (a), DMEM (b), and EBSS (c), and up to 2000 individual cells were analyzed. Images (2 h, DMEM/FCS) are shown (d). Scale bars: 20 *μ*m. The number of cells displaying GFP-WIPI-1 positive autophagosomal-like vesicles entrapping *S. aureus* HG001 was determined (e) ± SD (*n* = 2, each in duplicates).

**Figure 6:**
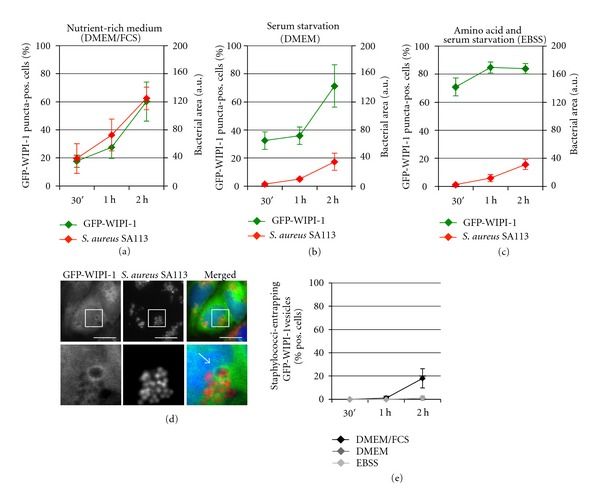
Pathogenic *S. aureus* SA113 induces GFP-WIPI-1 puncta formation and becomes entrapped in GFP-WIPI-1 positive autophagosome-like vesicles. According to Figures 4 and 5, GFP-WIPI-1 U2OS cells were infected with mCherry-expressing *S. aureus* SA113 in DMEM/FCS (a), DMEM (b), and EBSS (c) and analyzed (up to 2000 individual cells), representative images (2 h, DMEM/FCS) are shown ((d), scale bars: 20 *μ*m), and the quantification of cells displaying GFP-WIPI-1 positive autophagosomal-like vesicles entrapping *S. aureus* SA113 is presented (e) ± SD (*n* = 2, each in duplicates).

**Figure 7:**
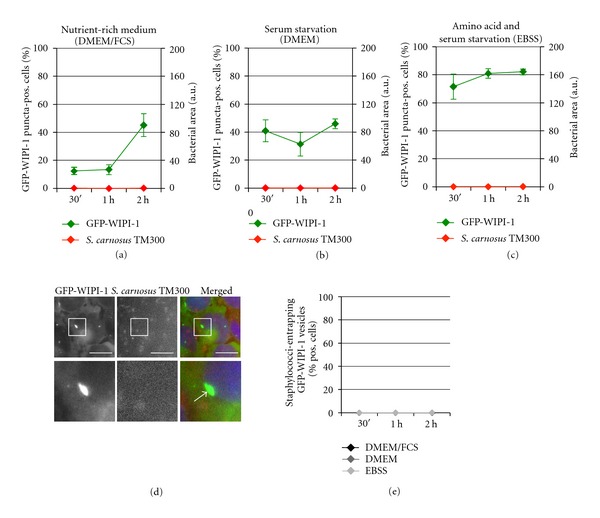
Apathogenic *S. carnosus* TM300 cells are not entrapped in GFP-WIPI-1 positive autophagosome-like vesicles. According to Figures 4–6, GFP-WIPI-1 U2OS cells were infected with mCherry-expressing *S. carnosus* TM300 in DMEM/FCS (a), DMEM (b), and EBSS (c) and analyzed (up to 2000 individual cells). Representative images (2 h, DMEM/FCS) are presented ((d), scale bars: 20 *μ*m). The number of cells with GFP-WIPI-1 positive autophagosomal-like vesicles entrapping *S. carnosus* TM300 is presented (e) ± SD (*n* = 2, each in duplicates).

In the following experiment, GFP-WIPI-1 expressing U2OS cells were infected for 0.5, 1 and 2 h with mCherry-expressing *S. aureus* USA300 (Figure 4, Supplementary Figure  2), HG001 (Figure 5, Supplementary Figure  3), or SA113 (Figure 6, Supplementary Figure  4) either in nutrient-rich medium (DMEM/FCS), serum-free medium (DMEM), or serum and amino acid-free medium (EBSS). Subsequently, fluorescence images (approximately 2000 individual cells per treatment) were automatically acquired and analyzed as described (Figure 3). Please note that the control experiments in Figure 1 were conducted in parallel to the experiments presented in Figures 4–7 hence provide the comparison for conditions without (Figure 1) and with (Figures 4–7, Supplementary Figures  2–5) Staphylococci.

As shown in Figure 1, under nutrient rich conditions (DMEM/FCS) the number of GFP-WIPI-1 puncta-positive cells is low (approximately 10%), reflecting cells that undergo basal autophagy. Interestingly, upon infection of GFP-WIPI-1 expressing U2OS cells with *S. aureus* USA300 in DMEM/FCS, a prominent increase of GFP-WIPI-1 puncta-positive cells (up to approximately 70% within 2 h of infection) was observed (Figure 4(a), in green). In addition, the number of GFP-WIPI-1 puncta per individual cell also increased upon *S. aureus* USA300 infection in DMEM/FCS (Supplementary Figure  6(B)). The elevated number of GFP-WIPI-1 puncta-positive cells and GFP-WIPI-1 puncta per cell correlated with an increase of intracellular *S. aureus* USA300 (Figure 4(a), in red). Using serum-free conditions either in the presence (DMEM, Figure 4(b), in red) or absence of amino acids (EBSS, Figure 4(c), in red), no increase of intracellular *S. aureus* USA300 was observed. However, infection of *S. aureus* USA300 in DMEM also resulted in an increase (up to approximately 70%) of GFP-WIPI-1 puncta-positive cells (Figure 4(b), in green), whereas *S. aureus* USA300 in EBSS (Figure 4(c)) did not trigger a further increase of the number of GFP-WIPI-1 puncta-positive cells when compared to EBSS treatment alone (Figure 1).

Next, we determined the number of cells displaying entrapped *S. aureus *USA300 within GFP-WIPI-1 positive autophagosome-like vesicles (Figures 4(d) and 4(e)). In line with the increased number of cells carrying intracellular *S. aureus *USA300 when nutrient-rich medium (DMEM/FCS) was used (Figure 4(a)), the number of cells with GFP-WIPI-1 positive autophagosome-like vesicles that entrap *S. aureus *USA300 (approximately 40%) also increased (Figure 4(e)). This was not observed by using DMEM or EBSS (Figure 4(e)). We also provide the control images corresponding to *S. aureus* USA300 infections using either DMEM or EBSS (Supplementary Figure  2).

The infection of stably expressing GFP-WIPI-1 U2OS cells with *S. aureus* HG001 in DMEM/FCS also triggered an elevation of GFP-WIPI-1 puncta-positive cells (up to 76%) (Figure 5(a), in green) and of GFP-WIPI-1 puncta per cell (Supplementary Figure  6(C)). Again, the increased number of GFP-WIPI-1 puncta-positive cells correlated with an increased bacterial load (Figure 5(a), in red) and the increase in the number of cells displaying GFP-WIPI-1 positive autophagosome-like vesicles entrapping *S. aureus* HG001 (approximately 40%) (Figures 5(d) and 5(e)). Also in this case, this feature was not observed by using DMEM or EBSS (Figure 5(e)), but DMEM conditions still triggered an increase of GFP-WIPI-1 puncta formation (Figure 5(b), Supplementary Figure  6(C)) when compared with control setting (Figure 1, Supplementary Figure  6(A)). Control images corresponding to *S. aureus* HG001 infections using either DMEM or EBSS are also provided (Supplementary Figure  3).

Next, we employed the *agr*-deficient *S. aureus* strain SA113 and infected stably expressing GFP-WIPI-1 U2OS cells. Clearly, upon infection in DMEM/FCS the number of GFP-WIPI-1 puncta-positive cells increased over time to up to 60% (Figure 6(a), in green), which correlated with an increasing bacterial load (Figure 6(a), in red). See also the increased number of GFP-WIPI-1 puncta per cell upon *S. aureus* SA113 infection in DMEM/FCS (Supplementary Figure  6(D)). In contrast to the effect of the employed *agr*-positive *S. aureus* strains USA300 (Figure 4) and HG001 (Figure 5), the number of cells displaying *S. aureus* SA113 entrapped in GFP-WIPI-1 positive autophagosome-like vesicles was prominently lower (approximately 18%) (Figures 6(d) and 6(e)). However, the presence of *S. aureus* SA113 in DMEM also triggered an increase of GFP-WIPI-1 puncta-positive cells (Figure 6(b)) when compared to control settings (Figure 1), whereas in EBSS no further elevation was achieved (Figure 6(c)), and in both cases, cells did not display entrapped *S. aureus* SA113 (Figure 6(e)). Control images of *S. aureus* SA113 infections with either DMEM or EBSS are also provided (Supplementary Figure  4).

### 3.4. Apathogenic *Staphylococcus carnosus* TM300 Cells Were Not Entrapped in Intracellular GFP-WIPI-1 Positive Autophagosome-Like Vesicles

In contrast to the pathogenic *S. aureus* strains (see above), infection of stably expressing GFP-WIPI-1 U2OS cells with the apathogenic *S. carnosus *TM300 did not result in an invasion of host cells in either of the used media (Figures 7(a)–7(c)). In line, GFP-WIPI-1 positive autophagosome-like vesicles were not induced (Figures 7(d) and 7(e)). Control images for *S. carnosus *TM300 in DMEM or EBSS are provided (Supplementary Figure  5). Interestingly, within 2 h of incubation with *S. carnosus *TM300 in DMEM/FCS, the number of GFP-WIPI-1 puncta-positive cells increased (approximately 45%) (Figure 7(a)) when compared to the control settings (Figure 1), which was not observed by using DMEM (Figure 7(b)) or EBSS (Figure 7(c)). However, the number of GFP-WIPI-1 puncta per individual cell did not increase upon infection of *S. carnosus *TM300 in DMEM/FCS (Supplementary Figure  6(E)) when compared to uninfected conditions (Supplementary Figure  6(A)).

### 3.5. Inhibition of PtdIns(3,5)P_2_ Production and Lysosomal Inhibition Increased the Number of WIPI-1 Positive Autophagosome-Like Vesicles Entrapping *Staphylococcus aureus*

**Figure 8:**
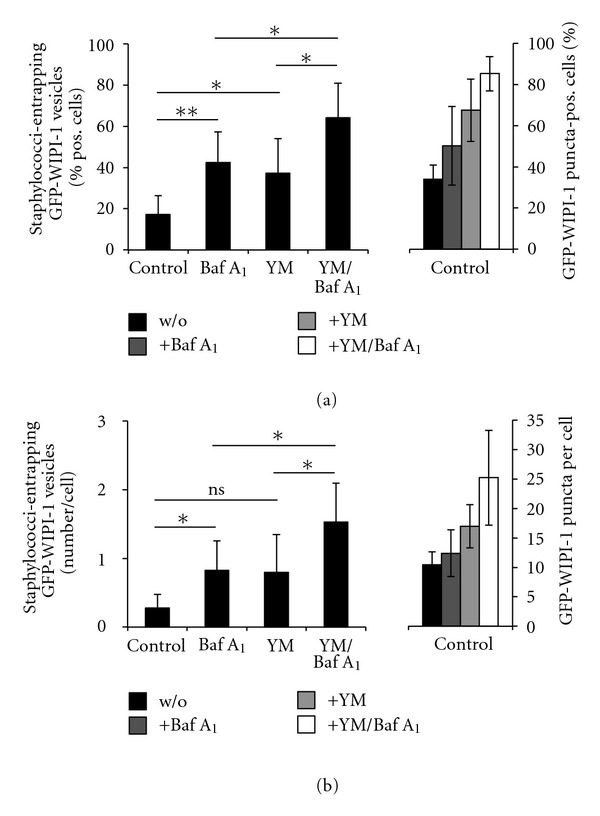
Bafilomycin A_1_ and YM201636 treatments increased the number of GFP-WIPI-1 positive autophagosome-like vesicles entrapping Staphylococci. GFP-WIPI-1 U2OS cells were infected with *S. aureus* USA300 in DMEM/FCS in the absence (control) or presence of 200 nM bafilomycin A_1_ (Baf A_1_), 800 nM YM201636 (YM), or with both (Baf A_1_/YM) for 2 h. Images were automatically acquired (not shown). The number of GFP-WIPI-1 puncta-positive cells ((a), right panel) and the number of GFP-WIPI-1 puncta per cell ((b), right panel) was determined. From 100 infected cells for each of the treatment condition, the number of cells displaying GFP-WIPI-1 positive autophagosomal-like vesicles entrapping *S. aureus* USA300 ((a), left panel) and the number of GFP-WIPI-1 autophagosomal-like vesicles entrapping *S. aureus* USA300 per cell ((b), left panel) was determined (*n* = 3). *P < 0.05, **P < 0.01, ns: not significant.

Next, we questioned whether pathogenic *S. aureus* cells entrapped in GFP-WIPI-1 positive autophagosomal-like vesicles are degraded in the lysosome. We employed the lysosomal inhibitor bafilomycin A_1_ (Baf A_1_) to block autophagosome/lysosome fusion events upon infection of GFP-WIPI-1 expressing U2OS cells with *S. aureus* USA300 in DMEM/FCS. Upon Baf A_1_ addition the number of cells harboring GFP-WIPI-1 positive autophagosomal-like vesicles entrapping *S. aureus* USA300 (Figure 8(a), left panel) significantly increased. And, the number of GFP-WIPI-1 positive autophagosomal-like vesicles per individual cell also significantly increased (Figure 8(b), left panel). In this situation (Figure 8(a), left panel; Figure 8(b), left panel) we found that the bacterial load did not significantly change (Supplementary Figure  7).

Further, during infection of GFP-WIPI-1 expressing U2OS cells with *S. aureus* USA300 in DMEM/FCS we employed YM201636 (YM), a specific PIKfyve inhibitor that blocks PtdIns(3,5)P_2_ production from PtdIns(3)P [24]. Upon YM treatment the number of cells harboring GFP-WIPI-1 positive autophagosomal-like vesicles (Figure 8(a), left panel) and the number of the vesicles per cell (Figure 8(b), left panel) significantly increased. Again, the intracellular bacterial load within the cells did not change (Supplementary Figure  7). Baf A_1_/YM cotreatment had an additive effect (Figures 8(a) and 8(b) left panels). The corresponding automated GFP-WIPI-1 puncta formation analysis is also provided (Figures 8(a) and 8(b) right panels).

### 3.6. Confocal and Electron Microscopy of Intracellular *Staphylococcus aureus* USA300

**Figure 9:**
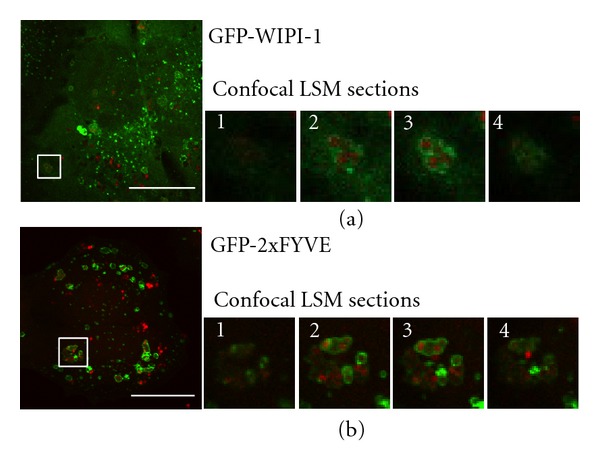
Confocal laser scanning microscopy of *S. aureus* USA300 infected GFP-WIPI-1 or GFP-2xFYVE expressing U2OS cells. GFP-WIPI-1 (a) or GFP-2xFYVE (b) expressing U2OS cells were infected with *S. aureus* USA300 for 2 h in DMEM/FCS. Representative images (*n* = 3) are shown. Magnifications display individual LSM sections (1–4). Scale bars: 20 *μ*m.

To achieve more image resolution, we infected GFP-WIPI-1 expressing U2OS cells with *S. aureus* USA300 in DMEM/FCS followed by confocal laser scanning microscopy (Figure 9(a)). Clearly, GFP-WIPI-1 positive autophagosome-like vesicles harbored multiple *S. aureus* USA300 cells and the analysis of individual confocal sections confirmed that these vesicles are found in the cytoplasm (Figure 9(a), 1–4).

It has been shown that *S. aureus* invading HeLa cells become sequestered in Rab7-positive endosomes [14]. As Rab7 marks late endosomes, we here used GFP-2xFYVE to visualize early endosomes. We used GFP-2xFYVE expressing U2OS cells for infection with *S. aureus* USA300 in DMEM/FCS. Indeed, we also found that *S. aureus* USA300 cells were entrapped in GFP-2xFYVE positive endosomes (Figure 9(b), 1–4).

**Figure 10:**
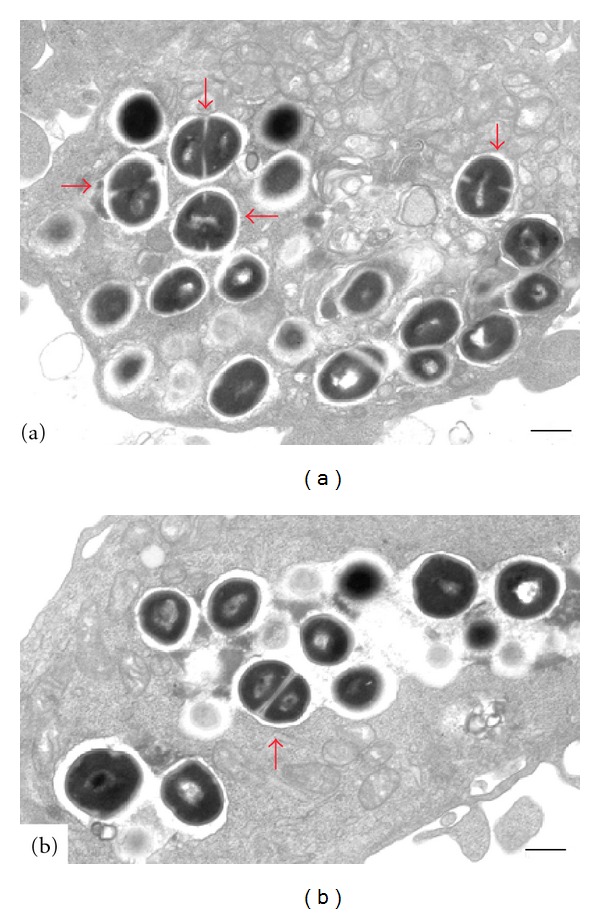
Electron microscopy of *S. aureus* USA300 infected GFP-WIPI-1 expressing U2OS cells. GFP-WIPI-1 U2OS cells were infected with *S. aureus* USA300 in DMEM/FCS followed by conventional electron microscopy. Either single *S. aureus* USA300 cells were found to reside within a vesicle (a), or multiple cells were found in enlarged vesicles (b). Red arrows indicate dividing Staphylococci. Scale bars: 500 nm.

 Further, by electron microscopy we found that intracellular *S. aureus* USA300 cells are entrapped in vesicles with a single *S. aureus* USA300 cell (Figure 10(a)), or in vesicles harboring multiple *S. aureus* USA300 cells (Figure 10(b)). In both cases, intracellular *S. aureus* USA300 cells showed clear signs of ongoing cell division (red arrows).

## 4. Discussion

Autophagy is considered an ancient eukaryotic pathway for cellular self-digestion that evolved with the endomembrane system [25]. As the endomembrane system provided an opportunity for invading pathogens to manipulate the host cell, it is further considered that the autophagic response to pathogen invasion may have also evolved as an early host defense program of eukaryotic cells [25, 26]. Interestingly enough, this hypothesis explains that (i) autophagy is in part a stochastic degradation pathway to clear the cytoplasm, thereby securing the functionality of both proteins and the endomembrane system, but is also (ii) a specific response triggered by certain stress exposures, such as pathogen invasion. In fact, the autophagic response to pathogen invasion has been identified because autophagy-related proteins (ATG) essential to the stochastic process of autophagy, such as Atg5 and LC3, have also been found to decorate membranes harboring intracellular pathogens and to be functionally involved in the cellular response to pathogens [4, 27]. Still, molecular mechanisms of autophagic responses to pathogen exposure are insufficiently understood.

Bacterial pathogens employ a variety of mechanisms to manipulate host cell membranes [28, 29]. Commonly, many bacteria interfere with the phosphoinositide metabolism that is often targeted by bacterial virulence factors [30]. Among the phosphoinositides, PtdIns(3)P is the essential variant for the forming autophagosomal membrane, hence it can be anticipated that PtdIns(3)P might commonly interconnect bacterial infection with the autophagic pathway. In fact, it has been shown that PtdIns(3)P is involved in the formation of Salmonella-containing vacuoles serving as a niche in host cells, and that PtdIns(3)P is targeted by *M. tuberculosis* to inhibit phagosome maturation [31]. Here, we addressed this question by investigating the process of *S. aureus* invasion of tumor cells.

A study by Schnaith and coworkers suggested a model that connected the autophagic response with *S. aureus* infection via the bacterial *agr*-virulence factor [14]. In this model, late phagosomes with (i) *agr*-positive *S. aureus* become entrapped in autophagosome-like vesicles, where *S. aureus* replicate and subsequently escape into the cytoplasm to promote host cell death, but (ii) *agr*-deficient *S. aureus* are subjected to lysosomal degradation [14].

We here provide evidence, that exposure of nonprofessional host cells (tumor cells) to Staphylococci stimulates the canonical WIPI-1 response at the onset of autophagy, which is to bind to PtdIns(3)P at the phagophore to foster the recruitment of downstream ATGs, such as Atg5 and LC3 [9, 32]. Interestingly, this response is attributable to the interaction of Staphylococci with the host cell membrane, as we found WIPI-1 to become stimulated upon both noninvasive and invasive Staphylococci. In line, WIPI-1 was also stimulated upon peptidoglycan treatment (data not shown). By further analyzing invasive *S. aureus* strains in this study, we identified new WIPI-1 positive autophagosome-like vesicles that entrapped multiple *S. aureus* particles. And, moreover, *agr*-positive *S. aureus* strains were more efficiently entrapped when compared to *agr*-deficient *S. aureus* cells. Our results demonstrate that WIPI-1, a principal PtdIns(3)P effector at the onset of stochastic, canonical autophagy, is also involved in selective engagement of the autophagic pathway, moreover underscored by the notion that Staphylococci prominently stimulated WIPI-1 in nutrient-rich conditions. And, our results demonstrate that *S. aureus* (i) stimulates autophagy and (ii) in addition, becomes entrapped in WIPI-1 positive autophagosome-like vesicles.

The most compelling explanation would be that WIPI-1 becomes stimulated upon *S. aureus *interaction with the plasma membrane, subsequently WIPI-1 positive phagophore membranes, for example, originated from the endoplasmic reticulum, are utilized to sequester *S. aureus* where bacterial replication occurs. In addition, we also found *S. aureus* particles sequestered in phagosomes, marked by the FYVE domain [33], which are intended for phagocytosis. Hence our results can be viewed as host cell response to *S. aureus*, critically involving PtdIns(3)P membranes that either serve as phagosome membranes, or that are utilized to further sequester *S. aureus*, thereby generating a replication niche. Evidence that bacterial replication occurs is given by our electron microscopy analysis showing dividing *S. aureus* cells within the sequestering vesicle. The importance of PtdIns(3)P-enriched membranes during sequestration of invading *S. aureus* is further emphasized by our finding that more WIPI-1 positive autophagosome-like vesicles entrap *S. aureus* cells when phosphorylation of PtdIns(3)P to PtdIns(3,5)P_2_ by PIKfyve was specifically blocked.

PtdIns(3)P-enriched membranes promote vesicle fusion with lysosomes. In line, FYVE domain marked phagosomes that carry *S. aureus* would be subjected to phagocytosis as suggested [14]. If WIPI-1 positive autophagosome-like vesicles entrapping *S. aureus* identified in this study would reflect cytoplasmic sequestration of invaded *S. aureus* with PtdIns(3)P-enriched WIPI-1 positive phagophores, the resulting autophagosome-like vesicles should become subjected to fusion with the lysosomal compartment, because they are enriched in PtdIns(3)P. But it was shown that lysosomal fusion is blocked upon *S. aureus* invasion [14]. To address this question we employed bafilomycin A_1_ to inhibit the functionality of the lysosomal compartment. Clearly, lysosomal inhibition significantly increased the number of WIPI-1 positive autophagosome-like vesicles harboring *agr*-positive Staphylococci. This demonstrates that nonprofessional host cells employ autophagy as a defense response with regards to *S. aureus* infection, in line with previous suggestions [34]. However, under some circumstances [14] bacterial replication and vesicle escape might override this cellular defense program.

## Supplementary Material

Here, we show the appearance of canonical and infection-induced GFP-WIPI-1 positive membranes upon *S. aureus* HG001 infection after 2h of infection in either DMEM/FCS, DMEM or EBSS medium (Supplementary Figure 1). Further, Staphylococci (*S. aureus* USA300, *S. aureus* HG001, *S. aureus* SA113, *S. carnosus* TM300) infected cells upon 2h of infection in DMEM and EBSS medium are shown (Supplementary Figures 2 – 5). In addition, the quantification of the GFP-WIPI-1 puncta per cell in uninfected cells and upon infection with different Staphylococci strains (*S. aureus* USA300, *S. aureus* HG001, *S. aureus* SA113, *S. carnosus* TM300) over time (30' -2h) is presented (Supplementary Figure 6). Finally, the bacterial load of *S. aureus* USA300 infected cells upon lysosomal and /or PIKfyve inhibition is presented (Supplementary Figure 7).

## Figures and Tables

**Table 1:** Bacterial strains used in this study.

Bacterial strain	Relevant properties	Relevant genotype	Reference
*S. aureus* USA300	Pathogenic, community-associated methicillin-resistant *S. aureus* (CA-MRSA)	*ag* *r* ^+^	[16]
*S. aureus* HG001	Pathogenic, methicillin-sensitive *S. aureus* (MSSA)	*ag* *r* ^+^	[17]
*S. aureus* SA113	Pathogenic, methicillin-sensitive *S. aureus* (MSSA)	*ag* *r* ^−^	[18]
*S. carnosus *TM300	Apathogenic, food grade staphylococcal species		[19]
