# Supplementary figures and images for: WIPI-1 Positive Autophagosome-Like Vesicles Entrap Pathogenic Staphylococcus aureus for Lysosomal Degradation
Source: Int J Cell Biol. 2012 Jul 9;2012:179207. doi: 10.1155/2012/179207 (PMC3399381; doi:10.1155/2012/179207)

DMEM/FCS

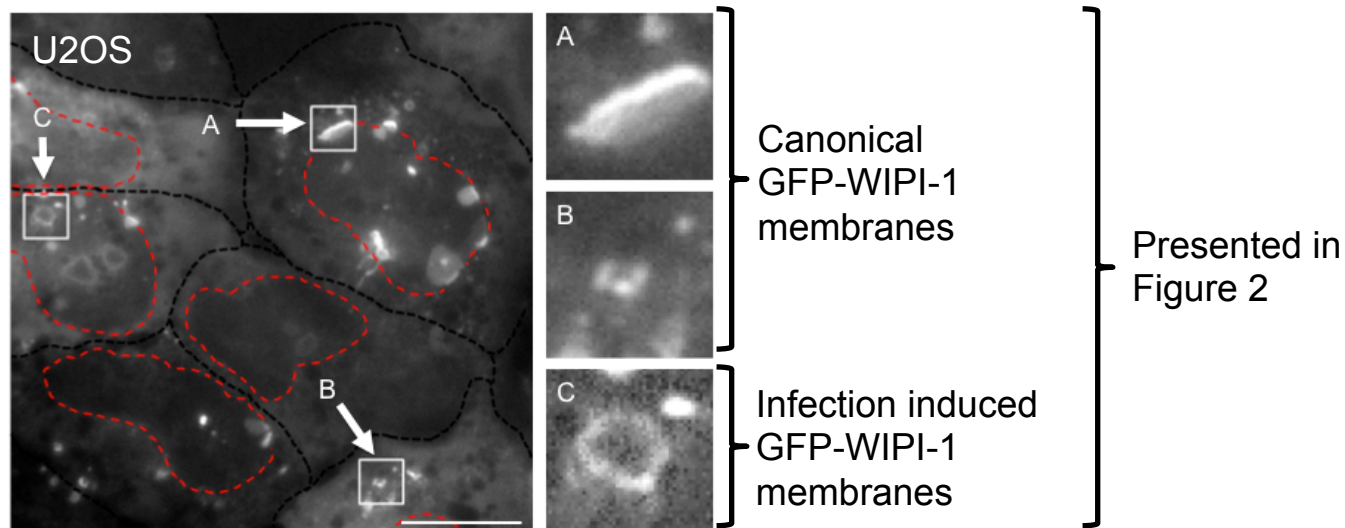

DMEM

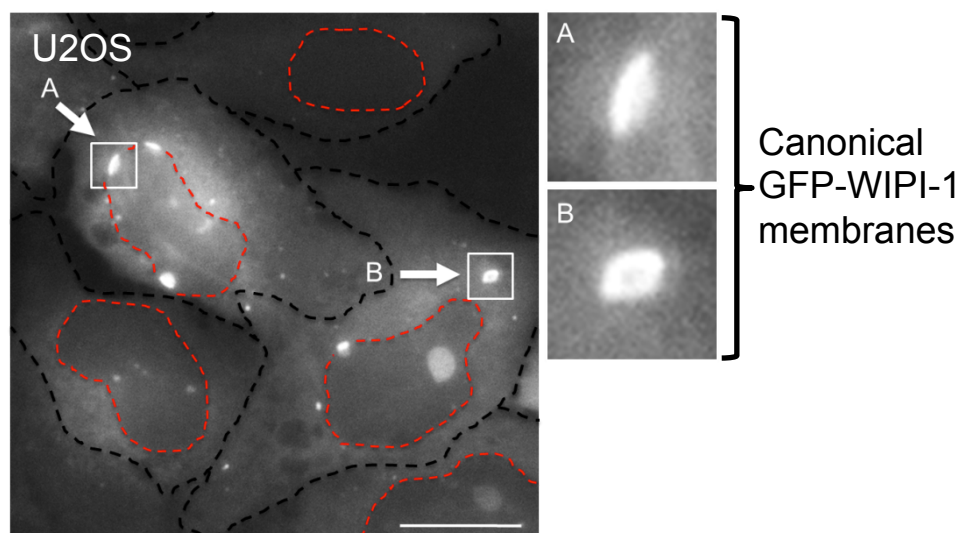

EBSS

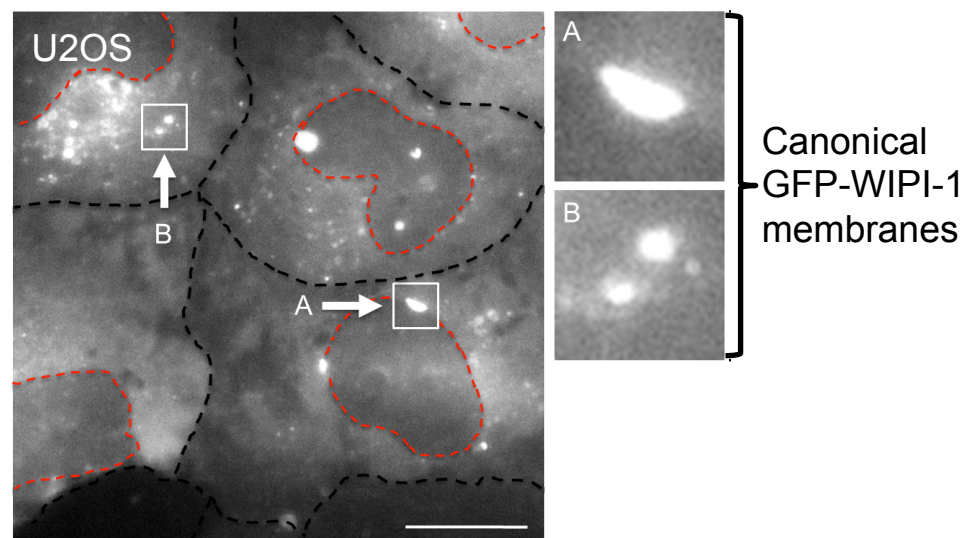

**Suppl. Figure 1**

Supplement: Supplementary file 1 — Here, we show the appearance of canonical and infection-induced GFP-WIPI-1 positive membranes upon S. aureus HG001 infection after 2h of infection in either DMEM/FCS, DMEM or EBSS medium (Supplementary Figure 1). Further, Staphylococci (S. aureus USA300, S. aureus HG001, S. aureus SA113, S. carnosus TM300) infected cells upon 2h of infection in DMEM and EBSS medium are shown (Supplementary Figures 2 – 5). In addition, the quantification of the GFP-WIPI-1 puncta per cell in uninfected cells and upon infection with different Staphylococci strains (S. aureus USA300, S. aureus HG001, S. aureus SA113, S. carnosus TM300) over time (30' -2h) is presented (Supplementary Figure 6). Finally, the bacterial load of S. aureus USA300 infected cells upon lysosomal and /or PIKfyve inhibition is presented (Supplementary Figure 7). [file 179207.f1.pdf]

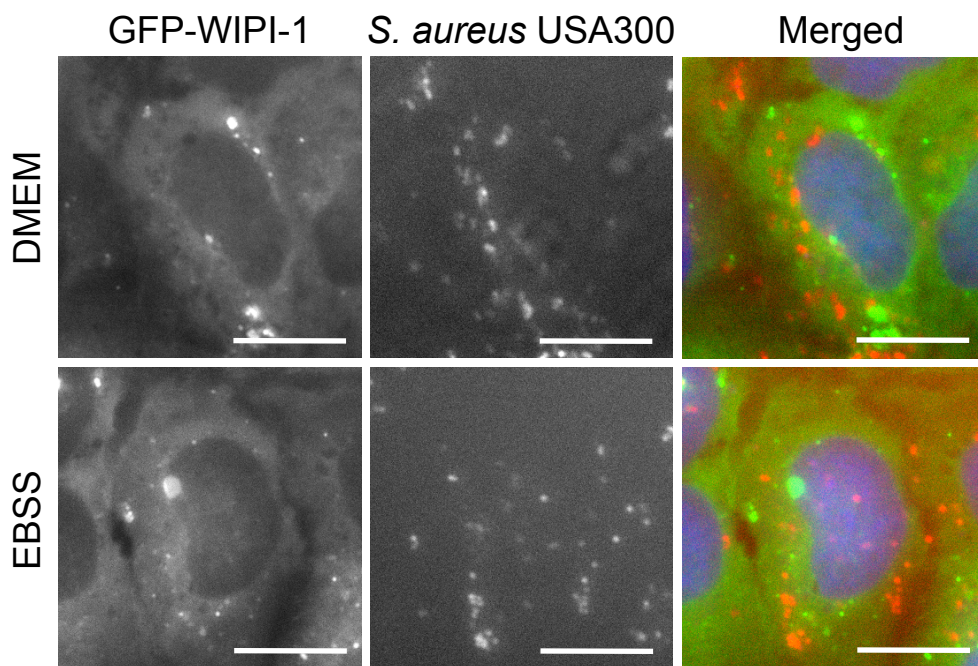

**Suppl. Figure 2**

Supplement: Supplementary file 2 [file 179207.f2.pdf]

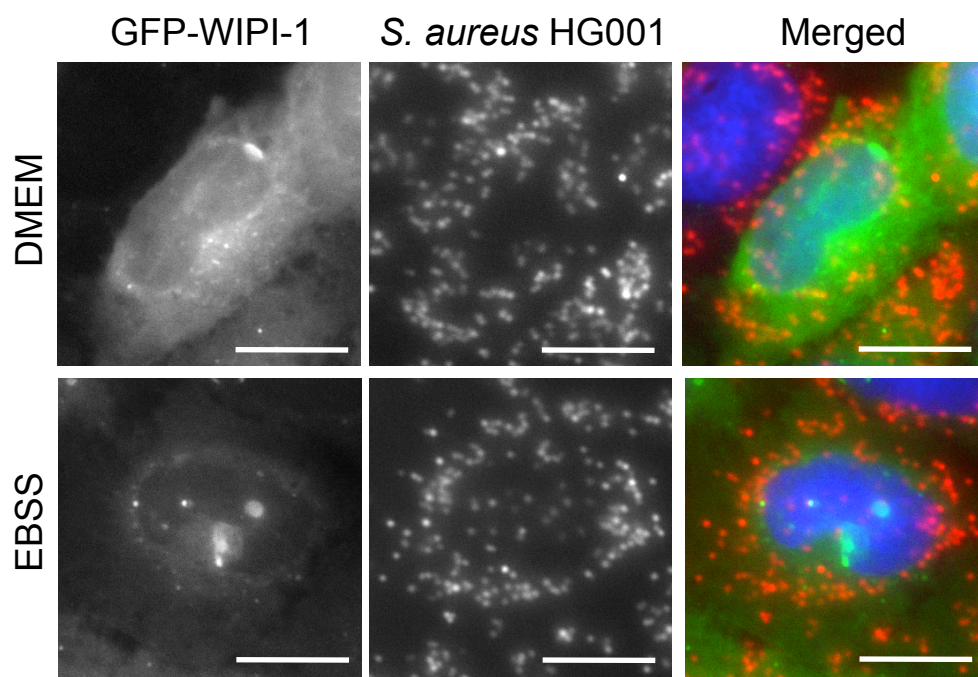

**Suppl. Figure 3**

Supplement: Supplementary file 3 [file 179207.f3.pdf]

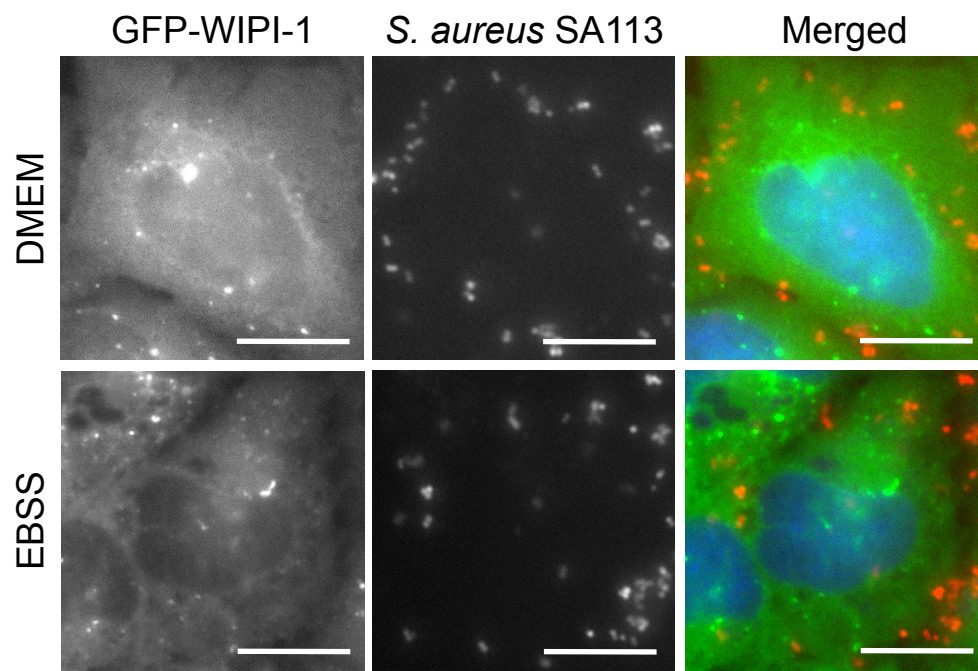

**Suppl. Figure 4**

Supplement: Supplementary file 4 [file 179207.f4.pdf]

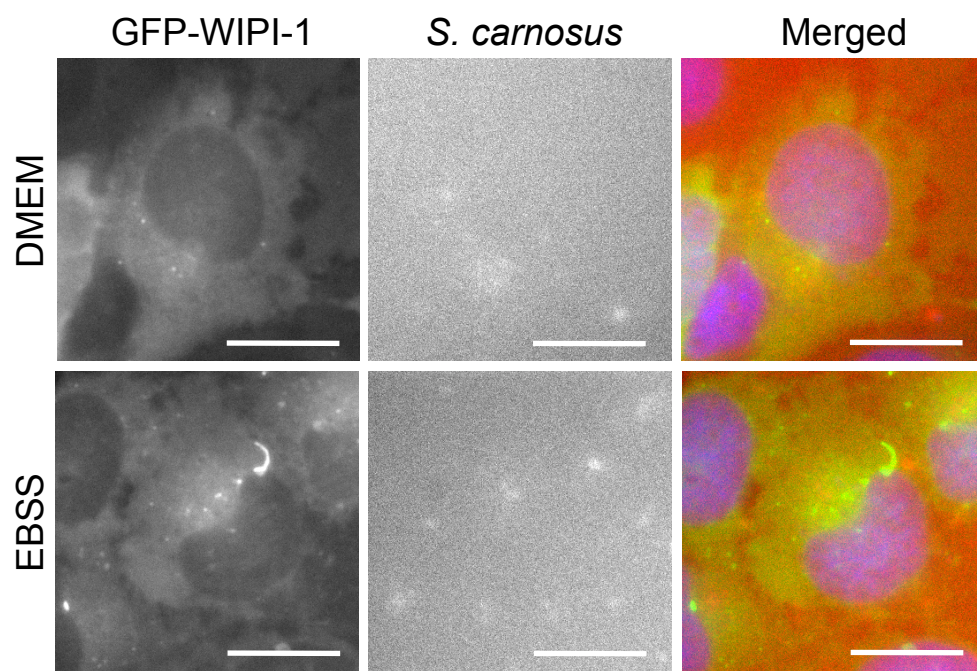

**Suppl. Figure 5**

Supplement: Supplementary file 5 [file 179207.f5.pdf]

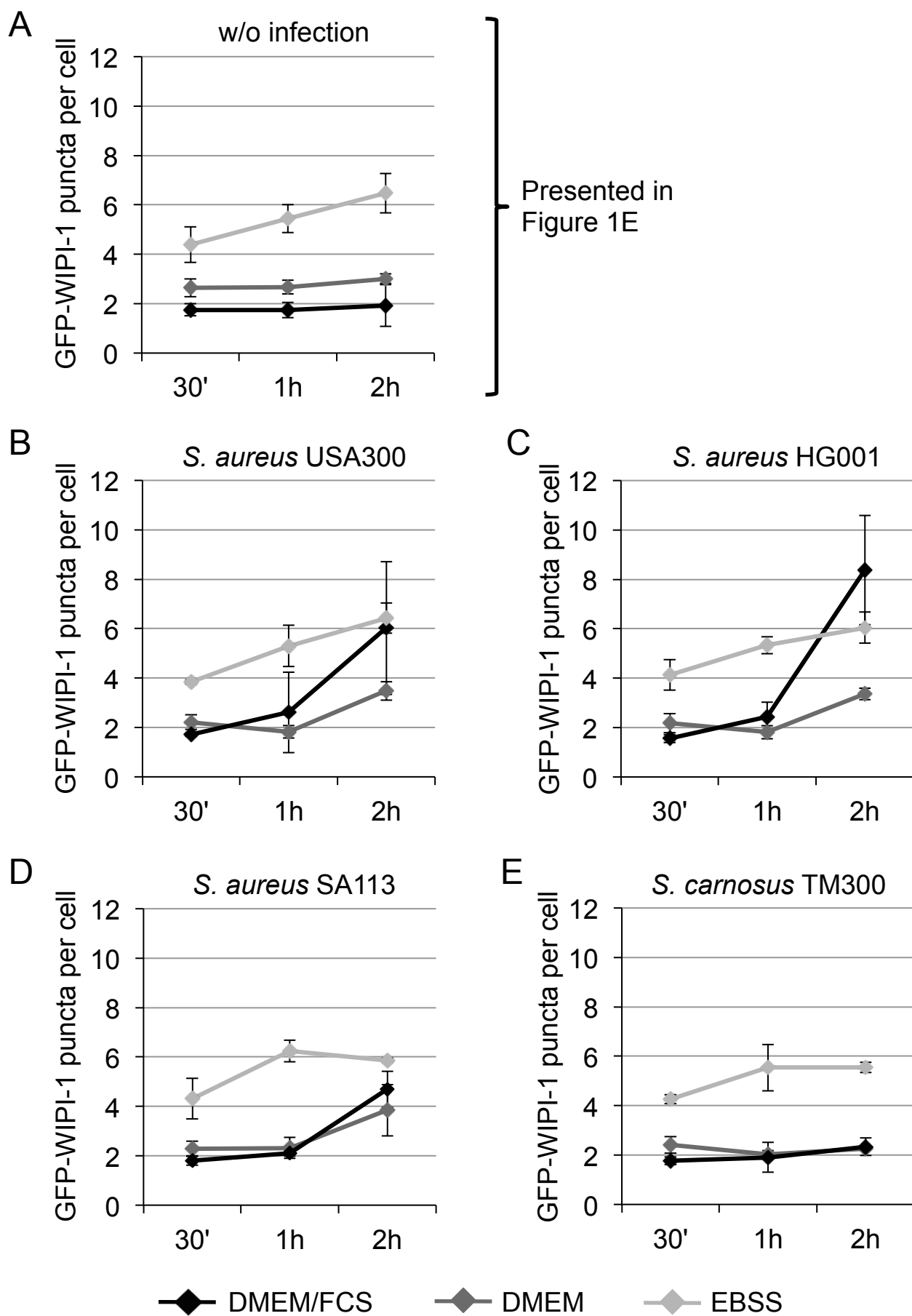

**Suppl. Figure 6**

Supplement: Supplementary file 6 [file 179207.f6.pdf]

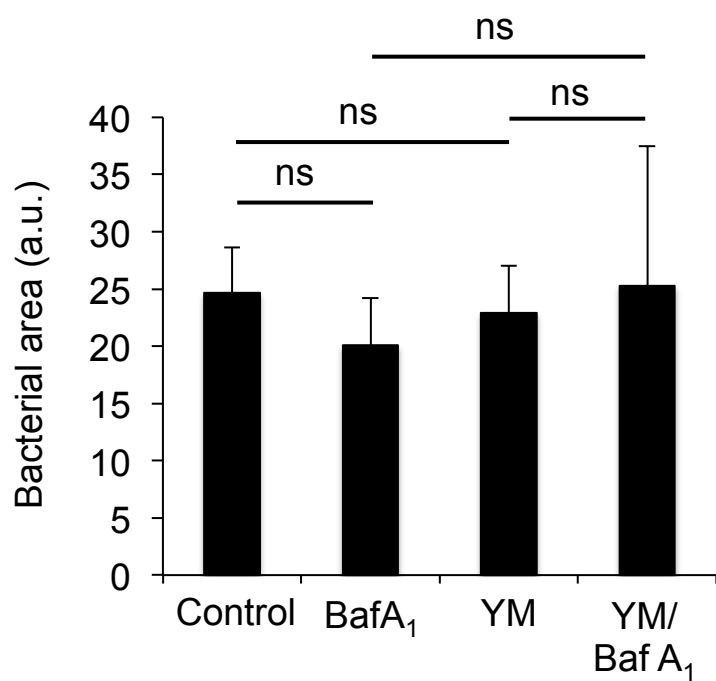

**Suppl. Figure 7**

Supplement: Supplementary file 7 [file 179207.f7.pdf]
